# Towards a Biologically Defined Diagnosis: Incorporating Pathophysiological Measures Into Parkinson’s Disease Clinical Criteria

**DOI:** 10.1155/padi/2703114

**Published:** 2026-02-25

**Authors:** Angus McNamara, Laura M. Carr, Irina Baetu, Mark Jenkinson, Lyndsey Collins-Praino

**Affiliations:** ^1^ School of Biomedicine, University of Adelaide, Adelaide, South Australia, Australia, adelaide.edu.au; ^2^ School of Psychology, University of Adelaide, Adelaide, South Australia, Australia, adelaide.edu.au; ^3^ Australian Institute for Machine Learning, School of Computer and Mathematical Sciences, University of Adelaide, Adelaide, South Australia, Australia, adelaide.edu.au; ^4^ South Australian Health and Medical Research Institute, Adelaide, South Australia, Australia, sahmri.com

## Abstract

Parkinson’s disease is the second most common neurodegenerative disorder worldwide, as well as being the fastest‐growing neurological disorder. Furthermore, PD corresponds to a significant burden on those diagnosed, their caregivers and healthcare systems, highlighting the critical need for early and accurate diagnosis. Effective diagnosis is essential not only for timely intervention but also for the development of disease‐modifying treatments, which are currently unavailable for PD management. Historically, PD diagnosis and characterisation was heavily reliant on clinical presentation, which are only present after significant neurodegeneration has already occurred. Because of this, there is a consensus amongst the scientific community to transition away from clinical features and instead redefine PD diagnosis and staging based on biological presentation. This review discusses historical developments in clinical diagnostic criteria for PD as well as the role the recently developed frameworks such as SynNeurGe and the Neuronal alpha‐Synuclein Disease–Integrated Staging System (NSD‐ISS) will play in the further advancements of diagnostic practices. Furthermore, substantial research efforts into the pathobiology of PD have led to the development of novel in vivo assessments capable of detecting critical biomarkers of PD. Specialised imaging modalities, particularly nuclear imaging and magnetic resonance imaging, and biomarkers of α‐synuclein pathology demonstrate high sensitivity and specificity for not only early diagnosis but also differential diagnosis between other parkinsonisms. Beyond diagnostic reforms, it is also important to identify markers that could serve as indicators of clinical course to aid in tailoring personalised treatment strategies. Therefore, this review also summarises key pathobiological hallmarks of PD beyond α‐synuclein pathology, namely, dopaminergic denervation, copathologies and underlying indicators of neurodegeneration, such as iron deposition and neuroinflammation. Furthermore, strategies to assess such pathologies and their potential utility in the current paradigm shift towards biological characterisation are discussed.

## 1. Introduction

Parkinson’s disease (PD) is the second most common neurodegenerative disease worldwide [1]. From 1990 to 2015, the number of individuals diagnosed with PD increased by 118%, with a worldwide prevalence of 6.2 million [2]. Notably, as of 2019, this increased again, rising to approximately 8.5 million confirmed PD cases worldwide, representing a 37% increase, within just a 4‐year period [3]. Concerningly, the prevalence of PD is expected to exceed 12 million cases by 2040 [4], making it the most rapidly growing neurological disorder in the world [2]. It should be noted, however, that such increases may not reflect a true increase in incidence, but instead may be in part due to other factors, such as increased vigilance and awareness of PD, as well as changes to diagnostic criteria. Nevertheless, Bloem and colleagues noted that while improved diagnostic accuracy by experienced clinicians partially explains the rise in incidence, this alone cannot explain why the age‐adjusted prevalence of PD is growing at a faster rate than other neurological disorders [5].

The rise in the prevalence is of major concern, as PD corresponds to a considerable economic burden, which is projected to surpass $79 billion in the United States alone by 2037 [6]. Therefore, earlier and more accurate diagnosis of PD is critically needed for effective therapeutic management. Most recently, an international conversation has been sparked following proposals to classify PD, and other alpha‐synuclein (α‐syn) diseases, based on their biological presentation [7, 8]. Important questions have been raised regarding the validation of pathobiological markers, particularly how well they correlate with the progression of clinical symptoms of PD [9]. Thus, the current review aims to explore recent advances in the field to assess the pathobiology of PD in vivo, with a particular focus on the sensitivity and specificity of such measures for early diagnosis and distinguishing PD from other forms of parkinsonism and for predicting the symptom trajectory during the course of the disease.

## 2. Evolution of Diagnostic Criteria in PD

The United Kingdom Parkinson’s Disease Society Brain Bank (UKPDSBB) criteria were developed in 1988, acting as the first official diagnostic criteria for PD. Prior to this, the diagnosis was reliant on the clinical judgement of cardinal symptoms and their progression, rather than a standardised diagnostic procedure [10]. While these criteria advanced the diagnosis considerably, highlighting bradykinesia as a core feature of PD [11] and incorporating levodopa responsiveness to distinguish PD from other parkinsonisms [12], they are associated with only a 76% autopsy‐confirmed diagnostic accuracy [13].

Following implementation of the UKPDSBB criteria, Gelb and colleagues proposed a new set of clinical criteria in 1999 [14]. Like the UKPDSBB criteria, Gelb’s clinical criteria were also primarily based on the presentation of cardinal motor features, including an individual presenting with at least two of the following features: resting tremor, bradykinesia, unilateral onset or muscle rigidity, as well as a positive levodopa response [14]. A key distinction between the UKPDSBB criteria and Gelb’s criteria was the introduction of three levels of diagnostic confidence: (1) clinically possible, (2) clinically probable and (3) clinically definite, adapted from previous attempts to redefine diagnostic criteria, albeit with limited traction [15, 16].

A direct comparison between UKPDSBB and Gelb’s criteria for diagnostic validity is limited, with, to our knowledge, only one study directly comparing the two [17]. This study determined that both criteria performed comparably for possible and probable PD (UKPDSBB: 92% positive predictive value, 25% negative predictive value; Gelb’s: 93% positive predictive value and 14% negative predictive value) [17]. However, sensitivity was higher using UKPDSBB criteria (90%), compared to both Gelb’s clinically possible PD (87%) and probable PD (72%), with both sets of criteria demonstrating low specificity (30%–40%) [17]. Low sensitivity may reflect broad spectrum features seen commonly across parkinsonisms, rather than features specific to PD, thus resulting in a potentially high rate of false‐positive diagnoses.

A major critique of both the UKPDSBB and Gelb diagnostic criteria is the heavy focus on motor features to provide a PD diagnosis. In support of this, Alder and colleagues determined that using such motor symptom‐based criteria, clinical diagnosis of PD yielded only an 88% sensitivity and 68% specificity for pathologically confirmed PD [18]. This highlights the need to incorporate other features of PD into diagnosis, in order to better distinguish PD from other forms of parkinsonism. In response to this, in 2015 the Movement Disorders Society (MDS) task force proposed a revised set of clinical criteria, known as the MDS‐PD criteria [19]. While designed for use in clinical research, standardisation and consequent reproducibility across sites and centres makes these suitable to guide clinical diagnosis [19].

Like past criteria, motor features are still a central feature of PD diagnosis, including the diagnosis of parkinsonism based on three cardinal motor manifestations—bradykinesia, plus either muscle rigidity or resting tremor (4–6 Hz). [19]. Once parkinsonism has been established, MDS‐PD criteria are used to determine a certainty level—either clinically established PD (based on the absence of absolute exclusion criteria, the presence of at least two supportive criteria and no red flags) or clinically probably PD (based on the absence of absolute exclusion criteria and the presence of red flags that are counter‐balanced by supportive criteria). Notably, supportive criteria include several ancillary diagnostic tests for nonmotor symptoms, including olfactory loss (adjusted for age and sex) and metaiodobenzylguanidine (MBIG) scintigraphy showing cardiac sympathetic denervation. Overall, MDS‐PD criteria demonstrate high utility for PD diagnosis, particularly when compared to other clinical criteria. In line with this, 94.5% of a cohort of 434 people with Parkinson’s (PwP) met criteria for probable PD, with 192 (88.5%) non‐PD participants being correctly identified as non‐PD, using MDS‐PD criteria [20]. Furthermore, MDS‐PD criteria displayed a significantly higher accuracy than UKPDSBB criteria (92% vs. 86%, respectively), when compared against the gold standard, a neurologist with > 10 years’ experience in PD diagnosis.

Despite these advancements in diagnostic criteria, however, even the MDS‐PD criteria are not with their limitations. In particular, such criteria still largely rely on clinical judgement, which is heavily influenced by rater expertise, reflected by large variability in clinical ratings across practitioners [21]. This is highlighted in a systematic review and meta‐analysis of 20 studies aiming to determine diagnostic accuracy for clinical PD, 11 of which used pathological examination as the gold standard. Amongst these 11 studies, pooled diagnostic accuracy was 80.6% (95% CrI: 75.2%–85.3%). Nonexpert diagnosis corresponded to poorer accuracy (73.8%; 95% CrI: 67.8%–79.6%) compared to movement disorder experts, but even for movement disorders, diagnostic accuracy only ranged from 79.6% (95% CrI: 46%–95.1%) to 83.9% (95% CrI: 69.7%–92.6%) after follow‐up, with significant variability noted [22]. Similarly, a study conducted by Joutsa and colleagues found that of the 77 cases of idiopathic PD diagnosed by general neurologists, only 75.3% were confirmed to be PD following neuropathological examination, suggesting that PD is largely overdiagnosed in generalist settings [23].

This is of great concern, as findings imply that effective clinical diagnosis is reliant on specialist expertise, with a recent study highlighting that of 685,116 Medicare beneficiaries with a diagnosis of PD, only 9.1% consulted a movement disorder specialist at least once during the calendar year [24]. It is also important to note that this study was conducted in the United States, where there are generally more resources and access to specialists for PD compared to many other countries. Consequently, geographic disparities in accessibility are likely even more pronounced globally. This was emphasised by a survey aiming to scope how accessible specialist movement disorders training is globally, which reported that certified training only exists in a minority of European countries and was not reported in African regions including Egypt and Tunisia [25].

Such shortcomings may, in turn, influence the high rates of misdiagnosis seen in PD, with an estimated 20% of PD cases being misdiagnosed and, subsequently, mistreated [26]. This is further corroborated by a recent case study from China, highlighting significant misdiagnosis rates of approximately 25%, which, interestingly, were more prevalent in individuals who presented with rigidity or gait abnormalities, compared to resting tremor [27]. Rates of misdiagnosis may also be influenced by the heavy priority still placed on motor features to identify PD, with many of the classic motor symptoms of PD overlapping with those seen in other forms of parkinsonism, such as progressive supranuclear palsy (PSP) and multiple system atrophy (MSA) [28].

## 3. Moving Forward—A Biologically Defined Diagnosis

Given these limitations, a new approach is clearly warranted. One such approach is to incorporate biomarkers of pathological changes, which are present years, or even decades, prior to clinical symptom onset [29]. In line with this, in recent years, multiple efforts have been made to integrate biomarkers and neuroimaging into the diagnostic process for PD. To date, two types of nuclear imaging have been approved by the U.S. Food and Drug Administration (FDA) for use in clinical settings, namely, dopamine transporter (DaT) single photon emission computed tomography (SPECT) and fluorodopa (F‐DOPA) positron emission tomography (PET) scans in 2011 and 2019, respectively [30, 31]. Both imaging techniques assess the nigrostriatal system, with DaT SPECT assessing dopaminergic terminal density [32] and F‐DOPA assessing the uptake of a radioactively labelled form of levodopa [33]. Furthermore, DaT SPECT and F‐DOPA have demonstrated similar utility in clinical settings, with both being able to distinguish parkinsonism from healthy controls [34], as well as advanced PD from *de novo* cases [35]. Nevertheless, DaT SPECT appears to demonstrate higher promise on account of higher sensitivity and is considered to be the current gold standard [36]. It is important to note, however, that while such imaging modalities are evidence of dopaminergic impairment, this is not specific regarding aetiology and thus has limited utility for differential diagnosis [37, 38].

Beyond nuclear imaging, research has also been conducted using structural magnetic resonance imaging (MRI) sequences, particularly susceptibility‐weighted imaging (SWI) and neuromelanin (NM)‐sensitive MRI. Both sequences are optimised to assess PD‐relevant structures, such as nigrosome‐1, and display high diagnostic utility for differentiating PD from healthy controls [39, 40]. Of note, these imaging modalities also display utility in differentiating PD from similar neurological conditions that present substantial clinical overlap, including other parkinsonisms, such as PSP and MSA [41].

Most recently, strides have also been made in the detection of α‐syn, with the recently developed seed amplification assay (SAA) being of particular promise. In fact, the SAA not only demonstrates high sensitivity in distinguishing PD from healthy controls [42] but can even identify prodromal PD prior to the emergence of cardinal motor features [43], suggesting that it may be a critical tool for earlier diagnosis. Additionally, a recent cross‐sectional study exploring the efficacy of skin biopsies to assess phosphorylated α‐syn determined that such a marker was highly present in PD (as well as other synucleinopathies), relative to healthy controls [44]. Despite the potential such objective biomarker assessments present for diagnosis, however, this has not yet translated to changes in diagnostic guidelines that formally incorporate these measures. In fact, cardiac sympathetic denervation on ^23^I‐metaiodobenzylguanidine scintigraphy is the only laboratory test currently included as a diagnostic criterion within MDS‐PD criteria [19].

Nevertheless, in 2024, there was a major push internationally to redefine PD [8], as well as α‐syn disease more widely [7], based on its pathobiological presentation. Regarding PD specifically, Höglinger and colleagues have proposed a biological classification system using a three‐component system, SynNeurGe: (1) the presence/absence of pathological α‐syn in tissues/CSF, (2) evidence of neurodegeneration and (3) presence/absence of pathogenic gene variants. These, in turn, are linked to a clinical component related to PD [8].

In parallel to the SynNeurGe framework, Simuni and colleagues put forward the Neuronal alpha‐Synuclein Disease–Integrated Staging System (NSD‐ISS) for synucleinopathy [7]. This is a triaxial biological framework, S‐D‐G, which includes (1) the presence or absence of neuronal α‐syn (S), (2) evidence of dopaminergic deficiency (D) and (3) the presence or absence of pathogenic SNCA gene variants (G) [7]. These are then integrated into a seven‐stage model, which also accounts for clinical features and functional impairment, aiming to support early detection, biological staging and appropriate cohort stratification for clinical trials [7].

Importantly, such frameworks, by integrating biomarkers into diagnostic criteria, offer the potential to detect underlying pathology prior to clinical onset, potentially allowing for considerably earlier diagnosis during the prodromal or preclinical stages of PD. This has sparked widespread discussion within the field, including the publication of a viewpoint paper by the MDS, acknowledging the potential promise of biomarkers as exploratory outcomes, but highlighting the need for significant further validation, including correlation with metrics of disease progression [9]. Similarly, several leading researchers in the field have published editorials on the matter, including Clifford Jack, who acknowledged the parallels with similar efforts in Alzheimer’s disease in recent years [45], as well as several others, who highlighted the promise, but also potential challenges and concerns, associated with this shift [46–48]. While very much in its infancy, it is clear that such classification systems sets the stage for a potential paradigm shift in the field. Furthermore, using this classification system, it might be possible to track changes in such markers over time within cohorts with a known high risk of future PD development (e.g., those with a particular genetic mutation [49] or a history of exposure to a known risk factor, such as prior history of head injury or pesticide/herbicide exposure [50]), which could aid in earlier diagnosis of PD. The pathobiological basis of PD, however, is highly complex, and therefore, it is important to consider what aspects demonstrate the highest current promise for future biomarker development.

Overall, recent advances in biomarker research have set the foundation for emerging frameworks that aim to improve classification and staging of PD based on its biological presentation. While the said developments may have important implications for redefining the diagnosis and management of PD, it is important to acknowledge that it is early days and concerns have also been raised regarding such synucleinopathy frameworks [51, 52]. Therefore, this review primarily seeks to highlight promising biomarkers that could be utilised to enhance the proposed research criteria. Prior to biological frameworks being taken up in practice, a significant further validation of the current markers of promise, as well as integration of novel markers reflective of additional pathologies, is needed.

## 4. The Role of α‐Syn in PD

A key aspect of both the SynNeurGe and NSD‐ISS frameworks is the detection of the presence or absence of neuronal α‐syn. To understand the measurement of this key biomarker, however, it is first necessary to understand how the pathological aggregation of α‐syn occurs in PD. The protein α‐syn is comprised of 140 amino acids and possesses three distinct structural features: (1) an N‐terminal region (residues 1–60), (2) a central region, with a nonamyloid compartment prone to beta‐sheet formation (residues 61–95) and (3) a C‐terminal region (residues 96–140) [53]. While its function under normal physiological conditions is still a matter of debate, α‐syn is hypothesised to play a role in neurotransmitter release due to its presynaptic location and interactions with synaptic membranes [54]. Of note, α‐syn in its monomeric form is natively unfolded and without a defined structure [55]. Consequently, monomeric α‐syn is relatively unstable, primarily within the C‐terminal region [56]. When exposed to post‐transitional modifications, such as serine 129 phosphorylation, ubiquitination and oxidation (for review, see Ref. [57]), its inherently flexible structure demonstrates a propensity to undergo structural modifications, such as misfolding and aggregation (see review, [58]). Consequently, in the pathophysiology of PD, α‐syn monomers undergo several structural modifications, including oligomerisation, fibrillation and, eventually, Lewy body formation.

### 4.1. Oligomerisation

In PD, following post‐translational protein modifications, and given an imbalanced rate of production/clearance, resulting in an overproduction of α‐syn, monomeric α‐syn misfolds and begins to aggregate, forming intracellular soluble oligomers [59]. Oligomers vary in composition and size and are key hallmarks of PD [59], with the presence of soluble α‐syn aggregates demonstrated both in midbrain neurons in α‐syn transgenic mouse brains and in both PD and dementia with Lewy bodies (DLB) postmortem tissue [60].

Many converging lines of evidence suggest that α‐syn oligomers are the toxic species that drive neurodegenerative processes. For example, in *in vitro* models of mesencephalic dopaminergic neurons, exposure to extracellular oligomeric α‐syn promotes cellular dysfunction and death via tubulin polymerisation and mitochondrial dysfunction [61]. Interestingly, Emin and colleagues also found that aggregates of wild‐type α‐syn, smaller than 200 nm in length, promote neurotoxicity via inflammation and permeabilization in vitro [62]. Not only does this suggest that oligomeric α‐syn induces a cascade of neurotoxic events, but that the size of α‐syn aggregates contributes to their toxicity. Similar findings have also been reported in vivo, with lentivirus and adeno‐associated virus viral vector rat models of PD demonstrating that, following the injection of α‐syn variants into the substantia nigra (SN), oligomeric species are the key driver of SN *pars compacta* (SNc) degeneration via membrane disruption [63], resulting in dystrophic neurites and gliosis within the striatum [64].

Notably, not only do α‐syn oligomers display high levels of toxicity, but they also demonstrate seeding capacity, which is critical for the spread of Lewy pathology throughout the brain. It has been shown that α‐syn oligomers can be released from diseased neurons into the extracellular space, and consequently taken up by previously healthy neurons [65–68]. Oligomers then act as templates, promoting endogenous monomers to undergo misfolding and aggregation, perpetuating increased oligomer formation and contributing to the spread of Lewy body pathology throughout the brain. In fact, it is the seeding principle that the SAA acts upon—the presence of pathology α‐syn in a biological sample acts as a seed to trigger precipitation (nucleation) of monomeric α‐syn within a superconcentrated solution of recombinant α‐syn [69].

### 4.2. Fibrillation

During the nucleation and growth phase of oligomerisation, oligomers increase in size and elongate, due to the incorporation of β‐sheets, which stack together, resulting in the formation of protofibrils (see review, [70]). Compared to the flexible monomeric and oligomeric form of α‐syn, protofibrils display a more organised structure [70]. Protofibrils can undergo further structural changes, such as alignment with other protofibrils and increased β‐sheet content, corresponding to highly organised, insoluble mature fibril formation [71, 72].

Overall, fibrillar forms of α‐syn are regarded as a less toxic species than the oligomeric structure, with inhibition of fibrillation leading to the persistent presence of oligomeric species, with subsequently increased oxidative damage and cell death [73]. Similarly, α‐syn variants with a propensity for fibril formation display less toxicity than those that form oligomers [63]. In fact, it has been suggested that mature fibrils may yield neuroprotective benefits by encapsulating toxic oligomers [74]. Nevertheless, evidence suggests that fibrils may still contribute to neurodegeneration, at least to some degree, through various mechanisms, including the promotion of neuroinflammation [75] and impaired lysosomal function upon uptake [76].

### 4.3. Lewy Body Formation

Once mature fibrils are formed, they accumulate and aggregate with surrounding fibril structures, eventually leading to the formation of intracellular inclusions called Lewy bodies. It is important to note, however, that Lewy bodies are not purely comprised of α‐syn, with several other constituent proteins also present (Table 1).

**TABLE 1:** Additional proteins that contribute to the development of Lewy bodies in PD.

Protein	Function	Role in Lewy body formation
Ubiquitin	Tags proteins, such as α‐syn, for degradation by proteasomes [77]	The presence of ubiquitin may indicate the failure of the ubiquitin proteasome system to degrade toxic α‐syn aggregates [77]
Alpha B crystallin	Present in pathological conditions [78], a molecular chaperon that binds to misfolded proteins to stabilise them via preventing misfolding and aggregation [79]	Chaperone activity is proposed to be either ineffective or overwhelmed in PD [80]
Neurofilament light	A key component of the neurofilament network; it provides structural support to neurons, as well as axonal transport [81]	An emerging marker of neurodegeneration; its elevated presence indicates potential axonal damage or neuronal injury [82]
Tau	Microtubule‐associated protein, involved in stabilising neuronal microtubules [83]	The literature suggests that hyperphosphorylated tau coaggregates with α‐syn, suggesting overlapping pathologies between neurodegenerative disorders [84]

An emerging theory is that, similar to fibrils, Lewy bodies may, in fact, be a neuroprotective mechanism—a strategy to sequester the more toxic oligomeric forms of α‐syn [85]. In line with this, Olanow and colleagues have proposed that Lewy bodies are a dysfunctional aggresome, forming in response to cellular stress to facilitate clearance of toxic α‐syn species [86].

### 4.4. Braak’s Staging of Lewy Body Pathology

In 2003, Heiko Braak notably proposed a staging of brain pathology in idiopathic PD based on the stereotypical spread of Lewy neurite and Lewy body pathology throughout the brain [87]. This was based on postmortem analysis of PD brains, which highlighted a topographical spread of pathology, starting in the anterior olfactory nucleus and dorsal motor nuclei of the glossopharyngeal and vagal nerves and gradually spreading to less vulnerable cortical areas in later stages of the disease [87]. Importantly, this spread mirrors the clinical presentation of the disease.

While extremely influential within the field, however, the Braak staging model is not without contention for several reasons, ranging from the current lack of a marker to investigate Lewy body progression in vivo, to limitations inherent with *postmortem* investigation, such as its retrospective nature and potential lack of information on clinical presentation [88], to concerns that the staging model does not fully capture the development of PD in all cases (see Ref. [89] for review). This has led to calls to ensure that other pathological markers, such as neuronal loss and neuroinflammation, are considered, in addition to Lewy body pathology, when considering PD progression [90, 91] (for review, see Ref. [89]).

#### 4.4.1. Tools to Measure α‐Syn as a Biomarker in PD

To date, one of the major limitations in detecting α‐syn in PD is that there is no current PET tracer capable of measuring α‐syn pathology that is validated for clinical use (for review, see Ref. [92]). This is in large part because of the low density of α‐syn aggregates in PD, compared to the higher density of amyloid beta (Aβ) or hyperphosphorylated tau (p‐tau) in Alzheimer’s disease, which makes developing a PD‐specific tracer challenging [93]. This limitation may be overcome by the recent development of ^18^F ACI‐12589, which has shown promising in vitro target engagement and specificity for pathological α‐syn in postmortem tissue from both PD and MSA cases, as well as its ability to distinguish individuals with MSA from healthy controls and other neurodegenerative diseases (including other synucleinopathies) [94]. Similarly, other potential PET tracer candidates have recently been put forward, including ^18^F‐F0502B, which shows selective binding with α‐syn fibrils in vitro, as well as good brain permeability, fast brain washout and recognition of α‐syn aggregates in nonhuman primate models of PD [93]. To date, however, research into such compounds is still in the relatively early stages, and a significant further validation is required prior to such compounds being ready for translation into the clinic and routine diagnostic use.

Nevertheless, measuring α‐syn in CSF has provided critical insights into the presentation and progression of pathophysiology in PD. Across several studies, there is a consensus showing that CSF concentrations of α‐syn are significantly lower in PD compared to healthy controls [95–98]. Furthermore, longitudinal assessment within the Parkinson Progression Markers Initiative (PPMI) cohort demonstrated that, compared to the baseline, CSF α‐syn was significantly reduced at both 24‐ and 36‐month follow‐ups, suggesting that CSF α‐syn levels decrease as the disease advances. Nevertheless, while decreases were seen over time, these did not correlate with either longitudinal motor scores, as measured by the MDS‐UPDRS, or striatal DaT scans, suggesting that levels of CSF α‐syn may not be an accurate reflection of underlying dopamine neurodegeneration and hence disease progression [95]. Furthermore, a meta‐analysis of 34 studies concluded that levels of CSF α‐syn did not significantly differ between PD and other parkinsonisms and only yielded a sensitivity and specificity of 72% and 65%, respectively, for distinguishing PD from healthy controls [99]. This limits the diagnostic utility of CSF α‐syn in clinical practice, highlighting the need to explore alternative measures of α‐syn with higher sensitivity and specificity to PD.

The recent development of the α‐syn SAA provides one such promising tool that can be used to measure α‐syn in clinical populations. In support of this, a cross‐sectional study of 1123 individuals in the PPMI database reported that CSF α‐syn SAA was able to distinguish PD from healthy controls with high sensitivity (98.6%) [42]. In fact, this sensitivity extends beyond clinically recognised PD, as SAA has also shown promise in detecting prodromal PD, with SAA being detected in 86% of individuals with REM sleep behaviour disorder or olfactory impairment, two common prodromal symptoms of PD, showing a positive α‐syn SAA in CSF samples [42]. When comparing the presence of a positive amplification result across prodromal features, a positive α‐synuclein SAA was reported in 85% of RBD cases and 89% of individuals reporting hyposmia, suggesting that hyposmia may be the best clinical proxy for a positive SAA [42].

Importantly, Siderowf and colleagues highlighted heterogeneity in the SAA assay based on both clinical presentation and genetic subtypes. For example, compared to PwP with hyposmia, resulting in an α‐syn SAA sensitivity of 97.2%, SAA positivity was only reported in 63% of PD cases without reported olfactory deficits [42]. Furthermore, the authors reported that no associations were present between RBD scores and α‐syn SAA status, again suggesting the utility of hyposmia as a clinical proxy for a positive SAA [42]. Likewise, compared to individuals with sporadic PD, where 93.3% reported SAA positivity, carriers of LRRK2 variants demonstrated markedly lower rates (67.5%) [42]. Interestingly, disparate rates persisted even when considering clinical proxies of SAA positivity, namely, olfactory function [42]. While LRRK2 carriers were more likely to have a positive α‐syn SAA (89.9%) than those with normoxia (34.7%), this was still much lower than the rates seen in sporadic PD cases with hyposmia (98.6%) [42]. Such findings support continued assessment of how SAA positivity varies as a function of genetic variant. This is particularly important, given the role that consideration of pathogenic gene variants plays in both the SynNeurGe and NSD‐ISS frameworks.

Regardless, the α‐syn SAA shows great potential in not only providing higher diagnostic confidence than can be achieved using current clinical criteria but could also translate to earlier diagnosis and indeed enhanced recognition of risk within the prodromal period. Likewise, neuronally derived extracellular vesicle α‐syn (i.e., L1EV‐associated α‐syn) has also been proposed as a promising biomarker of PD [43]. In a recent cross‐sectional study of 576 individuals, serum L1EV α‐syn differentiated high‐risk participants (> 80% probability of developing PD) from low risk, as well as controls. Furthermore, in a cohort of 40 individuals who later developed PD and related dementia, serum samples were positive for L1EV α‐syn in more than 80% [43].

Excitingly, recent developments also raise the possibility of being able to detect α‐syn in multiple easily accessible biological samples, including measurement of oligomeric α‐syn in plasma [100] and saliva [101–103] and phosphorylated α‐syn deposits in the skin [104–106] and submandibular gland [107]. In support of this, an observational study in the Systemic Synuclein Sampling Study measured α‐syn within multiple tissues and biofluids in the same individuals (59 individuals with PD and 21 healthy controls). Interestingly, while CSF levels of total α‐syn showed low specificity (63.2%) for distinguishing PD from healthy controls, the specificity for α‐syn immunoreactivity in the skin and submandibular gland was much higher (92.9% and 100%, respectively), suggesting that this method may have enhanced diagnostic utility [108]. Nevertheless, the sensitivity of such measurements was very low (only 56.1% and 24.1%, respectively), suggesting that alternative measurement methods or different α‐syn targets (other than total α‐syn) may be needed [108]. In support of this, a recent cross‐sectional study of 428 participants found that the proportion of individuals with PD with a skin biopsy positive for phosphorylated, as opposed to total, α‐syn was 92.7%, compared to just 3.3% in healthy controls (although it should also be noted that positive biopsies were found in 98.2% of individuals with MSA, 96% of individuals with dementia Lewy bodies and 100% of individuals with pure autonomic failure, highlighting that additional methods are necessary to distinguish between synucleinopathies) [44].

**Figure 1:**
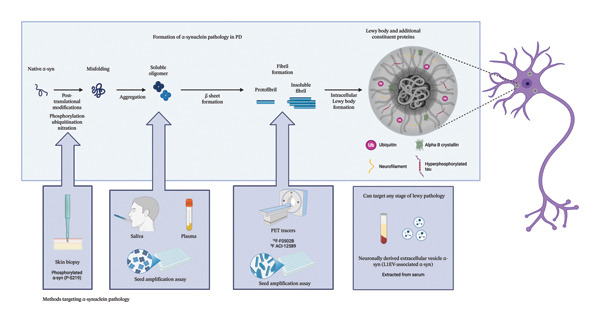
α‐syn conformational changes at various stages of Lewy pathology and techniques that have shown promise to assess.

Combining measures of α‐syn may also be necessary to enhance diagnostic accuracy. For example, a recent study in 38 individuals with PD and 24 healthy controls showed that combining measurements of soluble α‐syn oligomers within the saliva with skin biopsies for phosphorylated (P‐S129) aggregates distinguished PD from healthy controls with an AUC of 0.91 [109]. Taken together, continued advancements into the measurement of α‐syn levels in vivo provide hope for the development of a diagnostic/prognostic biomarker for PD, but further work must be done to validate such assays prior to their routine adoption into clinical practice. Furthermore, such work should incorporate recent advances in the field of α‐syn biology, including findings that different strains of α‐syn target distinct brain regions and cell types [110], to further enhance sensitivity and specificity. A summary of α‐syn conformational changes throughout various stages of Lewy pathology, as well as methods of assessing them, is outlined in Figure 1.

## 5. Co‐Pathology: Other Pathological Proteins Implicated in PD

A number of other proteins, including both hyperphosphorylated tau (p‐tau) and amyloid beta (Aβ), demonstrate interactions with α‐syn, suggesting synergistic effects between these proteins as drivers of PD pathology and emphasising the importance of co‐pathology in the disease (see review, [111]). For example, reduced cerebrospinal fluid (CSF) concentrations of Aβ^42^ are associated with more profound cognitive decline in PD, significantly predicting both delayed memory recall impairment over a 2‐year period [112] and general cognitive ability over a 1‐year period, even when adjusting for age, disease duration and baseline cognitive status [113]. Furthermore, assessment of CSF concentrations of α‐syn and Aβ^42^ in individuals with polygenic risk scores for PD found that increased risk scores were associated with reduced concentrations of both markers, with interactions between markers associated with cortical atrophy and reduced cognitive ability [114]. Beyond Aβ, a recent study by Chu and colleagues demonstrated that tau, rather than α‐syn aggregation, may mediate nigrostriatal dopamine neuron degeneration [115]. Despite this, however, few studies have examined the relationship between p‐tau and PD progression. One study reported that increased CSF levels of p‐tau significantly predicted more marked motor impairment over a 2‐year period [112]; however, evidence supporting its role in cognitive decline throughout PD is conflicting. For example, while a prospective cohort study of 45 PwP reported no associations between the CSF p‐tau concentration and cognitive decline over at least 1‐year follow‐up [113], more recent work, assessing plasma extracellular vesicles from 103 individuals with PD, showed that elevated tau levels within plasma extracellular vesicles at baseline corresponded to significantly greater decline in cognition, as well as motor function, over a 1‐year follow‐up [116].

### 5.1. Tools to Measure p‐Tau and Aβ as Biomarkers in PD

As highlighted above, several studies have measured CSF concentrations of Aβ and p‐tau in PD. In recent years, however, other, less invasive techniques have been utilised to assess PD copathologies in vivo, including the use of PET tracers.

#### 5.1.1. Aβ Ligands

In 2002, the ligand Pittsburgh Compound‐B (PiB) was developed, capable of detecting Aβ aggregates by binding to Aβ^42^ fibrils and protofibrils [117], with several other ligands developed since (see review, [118]). PET‐measured Aβ burden may be an important predictor of cognitive decline in PD. For example, a subset of idiopathic PD participants in the PPMI cohort (*n* = 25) underwent [^18^F]Florbetaben imaging, which showed a significantly higher Aβ burden in PD individuals compared to healthy controls (*n* = 30) [119]. Furthermore, in a model predicting global cognitive change over a 1‐year period, Aβ burden in the left gyrus rectus, the left anterior cingulate cortex and the right parietal cortex were significant predictors, explaining 49.5% of the total variance [119]. Such effects may be, at least in part, regionally specific. In line with this, ^18^F‐florbetapir assessment in 61 nondemented PwP found that Aβ levels in the posterior cingulate gyrus negatively correlated with verbal memory performance, whereas Aβ in the frontal cortex, precuneus and anterior cingulate gyrus negatively correlated with naming performance [120].

PET studies have also highlighted the potential utility of Aβ for predicting conversion to PD with mild cognitive impairment (PD‐MCI) and dementia (PD‐D). For example, of 25 PD‐MCI individuals undergoing [^18^F]flutemetamol scans, 32% demonstrated Aβ positivity and had significantly higher deposition in the right caudal and rostral middle frontal cortex, as well as the putamen bilaterally [121]. Furthermore, higher cortical Aβ burden was significantly associated with poorer executive function, although not global cognition or motor function [121]. Likewise, a study assessing 23 individuals diagnosed with PD‐D found that 17.5% of participants reported significant Aβ in the cortex, as well as significantly poorer performance on executive function tests [122]. Therefore, Aβ tracers may provide insights into which individuals are most susceptible to developing cognitive impairment throughout PD course. While not all PwP present with positive Aβ scans, in a recent study utilising [^18^F]FDDNP, it was found that individuals with PD‐D had significantly higher binding in lateral temporal regions compared to nondemented PD controls, which highly correlated with CSF Aβ concentrations [123]. Furthermore, higher Aβ deposition in the lateral temporal cortex was associated with more rapid cognitive decline over an 18‐month period [123], highlighting [^18^F]FDDNP as a potential biomarker of dementia risk or conversion to PD‐D.

#### 5.1.2. Tau Ligands

While PD is not considered to be a tauopathy, as outlined above, several lines of evidence implicate synergisms between α‐syn and tau pathology as drivers of PD development [84]. Tau PET imaging has previously demonstrated significant utility for enhancing diagnosis in AD, with the tracer ^18^F‐flortaucipir approved for clinical use for AD diagnosis in 2020 [124]. Similarly, tau imaging may provide a promising avenue to enhance PD diagnostics. In line with this, studies exploring tau ligands have demonstrated potential utility for distinguishing PD from other disorders. In a multicentre study assessing PwP (*n* = 26) or PSP (*n* = 33), individuals with PD demonstrated lower bilateral [^18^F]‐flortaucipir uptake in the GPi, putamen, subthalamic nucleus, midbrain and dentate nucleus, relative to PSP. Of note, globus pallidus *interna* binding demonstrated the highest utility for distinguishing PSP from PD (AUC = 0.893) [125]. Similarly, a systematic review of 15 studies reported reduced tracer uptake in the same regions compared to PSP, as well as reduction in the frontal and occipital lobes relative to AD and the infratemporal and occipital lobe compared to DLB [126]. Therefore, regional‐specific binding patterns could aid in the differential diagnosis of PD from other neurodegenerative disorders.

Beyond diagnosis, tau imaging may also be a critical indicator of disease course, particularly for cognitive impairment and eventual dementia diagnosis. In line with this, a study assessing [^18^]F‐florzolotau in PD‐D (*n* = 10), PD (*n* = 9) and age‐matched healthy controls (*n* = 9) found that the cortical uptake was significantly higher in PD‐D compared to both other groups [127]. Furthermore, uptake within the occipital lobe in PD‐D significantly correlated with cognitive impairment, as measured by Mini‐Mental State Examination scores (*r* = −0.635, *p* = 0.0486) [127]. Therefore, assessing tau pathology may not only enhance differential diagnosis but could also be used to better track disease course in PD, as well as provide a more objective strategy for forecasting the risk of potential cognitive impairment or dementia.

## 6. The Role of Dopaminergic Neuron Loss in PD

Another key aspect of both the SynNeurGe and NSD‐ISS frameworks is the degeneration of dopaminergic neurons, primarily within the SNc (see review, [128, 129]). When 60%–70% of dopaminergic neurons within the SNc are lost, the cardinal motor features of PD arise, leading to clinical diagnosis [130]. Dopaminergic neurons also comprise a significant portion of the VTA, an area which also experiences neuronal death throughout PD, albeit to a lesser extent than the SNc [131]. In fact, the VTA is the primary dopaminergic input for the mesocortical [132] and mesolimbic [133] pathways, responsible for cognitive functions, working memory and executive function (mesocortical), as well as reward processing, motivation and reinforcement learning (mesolimbic). As a consequence of progressive degeneration of the SNc and VTA, dopaminergic input to such pathways is substantially reduced in PD, leading to alterations in BG circuitry [134]. This results in widespread impairment in functions associated with each of the affected pathways, including motor function (nigrostriatal pathway), cognitive and executive impairments (mesocortical) and impaired reward processing and motivation (mesolimbic) [134]. There is overlap between pathways and functional domains, with, for example, nigrostriatal deficits also being associated with changes to reward processing in PD [135]. Given the central role that dopamine plays in the clinical presentation of PD, effective tracking of dopaminergic changes in PD is critical.

### 6.1. Tools to Measure Dopamine Alterations as a Biomarker in PD

#### 6.1.1. DaT SPECT

In 2011, the FDA approved the use of DaT SPECT imaging, specifically ioflupane or [^123^I]‐FP‐CIT, as a tool to assist in PD diagnosis [30]. DaT SPECT works by assessing dopaminergic terminal density [32], which is substantially reduced in PD due to significant neuronal loss. Overall, DaT SPECT imaging possesses high sensitivity for identifying dopaminergic deficiency, with a systematic review of eight studies reporting that the nigrostriatal cell loss was detected with a sensitivity and specificity of 98% [136]. Furthermore, the use of DaT SPECT has been shown to correspond to improved diagnostic confidence, with a study of 57 individuals presenting with movement disorders determining that, in clinically diagnosed PD (*n* = 24), DaT imaging had a positive predictive value of 92%, as well as high interobserver agreement between scans (91%), supporting its reproducibility within clinical settings [137].

Importantly, DaT SPECT imaging demonstrates potential for differentiating PD from other conditions that overlap in their clinical presentation, which are not associated with degeneration of the dopaminergic system. For example, in individuals with essential tremor, while mild reductions were found in striatal DaT when compared to healthy controls, this was not as profound as that seen in PD [138]. In fact, DaT SPECT allows for differential diagnosis between essential tremor and PD with a specificity ranging from 97% to 100% [139]. Similarly, DaT scans have been shown to have utility for distinguishing between PD and drug‐induced parkinsonism [140], psychogenic parkinsonism [141] and, in at least a subset of cases, vascular parkinsonism [142]. Considering challenges and disparities in the ability to distinguish PD from such disorders clinically, DaT SPECT shows promise in improving diagnostic confidence beyond clinical expertise alone.

Studies exploring DaT SPECT have also reported associations between imaging findings and symptom presentation and progression in PD. In line with this, in 41 PwP, 123I‐FP‐CIT uptake in the caudate, putamen and overall striatum was negatively associated with disease severity, as measured by the UPDRS (*r* = −0.54, *p* = 0.0003), as well as disease duration (*r* = −0.67, *p* < 0.0001) [143]. Similarly, DaT has utility for tracking nonmotor symptoms, with a study of 76 PwP showing that not only was striatal uptake significantly lower in PD compared to 46 age‐matched healthy controls, but reduced DaT binding in the putamen corresponded to more severe anxiety, depression and overall affective outcomes [144]. Such effects may be regionally specific, with individuals in the PPMI cohort presenting with bilateral caudate impairment at baseline (22.5%) presenting with higher rates of cognitive impairment and depression compared to individuals with normal caudate DaT levels (51.6%) [145]. Furthermore, in longitudinal studies, DaT binding in striatal regions has been shown to decrease over time (∼4%–15% annually) throughout disease course, highlighting its sensitivity for capturing progressive dopaminergic loss in PD [144, 146]. In line with this, a previous postmortem study showed a significant correlation between striatal DaT imaging and neuron count within the SN [147]. It is important to note, however, that not all studies have supported such an association, with a more recent study proposing that reduced striatal DaT binding in PD may instead reflect axonal damage or changes in DaT expression, rather than nigrostriatal neuron loss [148].

#### 6.1.2. F‐DOPA PET

In 2019, the FDA approved the use of [^18^F]‐DOPA positron emission tomography (F‐DOPA PET) imaging to aid in early diagnosis of PD [31]. F‐DOPA PET is used to evaluate the nigrostriatal system by binding to dopaminergic neuron terminals [33]. Overall, F‐DOPA PET demonstrates high sensitivity in identifying dopaminergic deficiency in PD, with an early systematic review of 13 studies reporting that all studies found that a diagnosis of PD corresponded to significant reductions in striatal F‐DOPA uptake, with prospective studies also suggesting that F‐DOPA PET can capture reductions in uptake with disease progression [149]. Compared to DaT SPECT, the reductions in striatal activity found using F‐DOPA PET are consistently smaller [150], although it is important to note that studies comparing the two methods within the same individuals are quite limited [34, 36, 151, 152].

Altered F‐DOPA uptake of the nigrostriatal system demonstrates high utility in distinguishing parkinsonism from healthy controls. For example, profound deficiencies in several regions allows for determination of parkinsonism, including the posterior putamen and, to a lesser extent, the caudate nucleus [153]. This likely reflects regionally specific vulnerability within the SNc, whereby the ventrolateral SNc deteriorates earlier and more rapidly compared to the medial portion [130, 154], with these regions projecting to the putamen and caudate, respectively [155].

Beyond identification of parkinsonism, like DaT SPECT, F‐DOPA also shows potential in differential diagnosis. In a study comparing striatal F‐DOPA influx constants for 20 individuals with predominantly postural tremor (8 familial, 12 sporadic), 11 with predominantly resting tremor, 16 individuals with PD and 30 healthy controls, the striatal F‐DOPA uptake was normal in the familial essential tremor group and lower than normal in only two out of 12 of the sporadic cases [156]. Conversely, in the 11 resting tremor patients, the mean putamen F‐DOPA uptake was similar to that seen in the PD patients (51% of normal) [156]. Similarly, in a study of 13 individuals with severe drug‐induced parkinsonism, with 12 followed‐up to a median of 23.5 months, nine out of 12 participants presented with normal putamen F‐DOPA uptake [157]. Encouragingly, the pattern of uptake seen may also help to distinguish PD from other parkinsonian disorders, including MSA and PSP. In line with this, in people with PD, uptake in the posterior putamen is significantly impaired (45% of normal), while the uptake in the anterior putamen and caudate is relatively spared (62% and 84% of normal, respectively). Conversely, in patients with PSP, impairment of uptake was similar in both the anterior and posterior putamen, with caudate uptake also being significantly impaired. Levels of the impaired uptake in MSA were between those noted in PD and PSP [158]. Similarly, a study utilising discriminant function analysis found that the F‐DOPA uptake was able to successfully discriminate PD from patients with the Steele–Richardson–Olszewski syndrome, although was less successful in discriminating PD from MSA [159].

This is important, as the ability to distinguish between parkinsonian syndromes has implications for disease management. This was highlighted in a recent study in an Australian movement disorders clinic, where authors reported that upon reviewing 105 F‐DOPA PET scan results and patient records, provisional clinical diagnoses were changed in 37.9% of patients, with changes to clinical management made in 48.4% of cases [160]. Overall, such findings support the incorporation of F‐DOPA PET, alongside the current clinical criteria, to streamline diagnosis and management of PD.

Of note, F‐DOPA also may have utility for predicting symptom severity and progression (see review, [161]). In line with this, decreases in the F‐DOPA uptake have been shown to have a high negative correlation with patient rigidity and bradykinesia scores, but not the degree of tremor [162]. Similarly, PD MDS‐UPDRS motor ratings have been shown to be moderately correlated with striato‐occipital ratios (SORs) measured using F‐DOPA imaging in those with a mild motor symptom presentation [152], as well as with both motor UPDRS scores (*R* ^2^ = −0.38) and disease duration (*R* ^2^ = −0.49) in comparisons between *de novo* PD and advanced PD [35]. Interestingly, regional differences in the F‐DOPA uptake may have utility for predicting specific functional changes. In line with this, while both putamen and caudate uptakes correlated with PD severity, the former was more implicated in the locomotor disability [163]. Similarly, in a study in an advanced PD cohort (disease duration = 11.8 ± 4.5 years), the regional F‐DOPA uptake was associated with differential cognitive impairments [164]. Specifically, the reduced F‐DOPA uptake in the putamen was most associated with executive function (*r* = 0.44), but less so with memory (*r* = 0.26) and verbal fluency (*r* = 0.32), whereas the inverse was reported for the caudate (executive function: *r* = 0.37, memory: *r* = 0.41 and fluency: *r* = 0.44) [164].

#### 6.1.3. DaT PET

While DaT SPECT and F‐DOPA PET have been widely studied and are clinically utilised, a current area of growing research focus is DAT PET imaging. DAT PET offers several advantages over traditional DaT SPECT, including shorter acquisition times [165], higher resolution, better signal‐to‐noise ratio and better promise for multimodal target assessment [166]. To this end, several candidate tracers have been developed, as outlined in Table 2.

**Table 2:** PET radioligands that bind to DaT and are commonly assessed in the PD literature.

Tracer	Label
[^11^C]PE21 [167]	Carbon‐11
[^18^F]FECNT [168]	Fluorine‐18
[^18^F]FP‐CIT [169]	Fluorine‐18
[^11^C]β‐CFT [170]	Carbon‐11
[^18^F]LBT‐999 [171]	Fluorine‐18

DaT PET shows a similar utility to DaT SPECT and F‐DOPA in identifying parkinsonism. In line with this, a meta‐analysis of six 18F‐FP‐CIT studies, encompassing 779 individuals with PD and 124 healthy controls, found that PD corresponded to significant reductions in several regions affected in PD, including the caudate nucleus, anterior putamen and posterior putamen [172]. Furthermore, DaT PET also shows promise in improving diagnostic confidence and providing differential diagnosis from forms of parkinsonism without dopaminergic deficits. In a study of 272 PwP, 111 presented with a clinically uncertain diagnosis, however, focal DaT binding via [^18^F]‐FP‐CIT PET greatly improved sensitivity, specificity and accuracy (98.7%, 100% and 99.1%, respectively) compared to initial clinical diagnosis (54.4%, 50% and 53.2%, respectively) [173]. Similarly, the [^18^F]‐FP‐CIT PET striatal uptake was shown to be largely reduced in PD, although normal in essential tremor.

In fact, within the same study, a direct comparison of DaT SPECT and DaT PET using ^123^I‐FP‐CIT and [^18^F]‐FP‐CIT, respectively, found that while visual analysis of each modality did not affect diagnostic accuracy, semiquantitative analyses indicated that PET DaT demonstrated better contrast [174]. This was corroborated by a study of 16 individuals with a parkinsonian disorder, highlighting that a single [^11^C]‐PE2I scan could act as an alternative to dual examination with ^123^I‐FP‐CIT SPECT and [^18^F]‐FDG PET [175]. Despite promising findings supporting further investigation into DaT PET, however, studies directly comparing PET and SPECT DaT tracers are limited, warranting further research.

Beyond diagnostics, DaT PET also shows important associations with clinical features of PD, including disease staging and motor symptom severity. In a study of 75 PwP, the striatal uptake of [^11^C]C‐CFT yielded significant negative correlations with UPDRS Part Three total scores, Hoehn and Yahr scores and Hoehn and Yar staging [176]. Beyond motor features, the asymmetry index in the putamen and posterior putamen were also significantly associated with Mini‐Mental State Examination and UPDRS Part Two scores, respectively [176]. Similar findings were also reported in a cross‐sectional study of 38 PwP who underwent [^18^F]‐FP‐CIT PET, which found that uptake in basal ganglia structures was strongly correlated with UPDRS Part Three motor scores [177]. Furthermore, left caudate nucleus uptake positively correlated with axial or akinetic rigidity symptoms [177]. Considering akinetic rigid subtypes of PD are known to present with greater dopaminergic deficiency and more rapid disease progression relative to tremor‐dominant (TD) subtypes [178], regional DaT PET uptake patterns may therefore help to improve the forecasting clinical trajectory in PD. Overall, DaT PET shows promise not only for diagnosis and prognosis but also offers several advantages over DaT SPECT and F‐DOPA, warranting further research for potential integration into clinical practice.

#### 6.1.4. Limitations of Nuclear Imaging of the Dopaminergic System

Despite promise, dopaminergic system evaluation via SPECT or PET is not without limitations, namely, an inability to differentiate between PD and other neurodegenerative parkinsonisms associated with profound dopaminergic degeneration, such as MSA and PSP [37, 38]. For example, familial PSP patients exhibit significantly reduced F‐DOPA uptake in the striatum and orbitofrontal cortex bilaterally and right amygdala, making it impossible to distinguish idiopathic PD from PSP based on the F‐DOPA uptake alone [179]. Thus, it may be necessary to employ a multitracer approach [38]. Furthermore, a subset of individuals with a clinical diagnosis of PD, including responsiveness to levodopa therapy, present with normal DaT SPECT scans (i.e., scans without evidence of dopaminergic deficit or SWEDDs) [180, 181].

Furthermore, several limitations with nuclear imaging limit clinical efficacy, such as high costs, limited availability and challenges surrounding the production of tracers. It may therefore be beneficial to explore the incorporation of more cost‐effective and readily available imaging modalities, including recent advances in MRI.

#### 6.1.5. NM‐Sensitive T1 MRI

Historically, conventional T2‐weighted MRI has been used to detect degeneration in regions of interest (ROIs) known to be affected during PD, such as the SNc. For example, early clinical studies found significantly higher SNc iron content in PD compared to healthy controls [182, 183]. Such increased iron content was found to be proportional to disease severity, supporting the use of iron as measured by MRI as a potential biomarker of PD [183]. Despite this, however, conventional MRI is limited in its ability to image key brainstem nuclei and other changes to BG circuitry. Conventional T1 and T2 MRI images are not sufficiently detailed in such deep regions, due to unspecific contrast and insufficient spatial resolution to discriminate between such structures [184]. Additionally, SNc imaging with standard magnetic resonance sequences usually appears normal in early PD [184]. In recent years, however, several advances in MRI, particularly the development of NM‐sensitive T1 MRI, have made this an increasingly valuable tool in the diagnosis of PD.

NM is a by‐product of iron‐modulated oxidation of cytosolic dopamine not accumulated in synaptic vesicles within the midbrain [185]. The progressive loss of dopaminergic neurons throughout PD results in significant reductions in the overall dopamine content and, consequently, less NM [186, 187]. To this end, Sasaki and colleagues developed a T1‐weighted protocol using a 3‐Tesla magnet, aimed at enhancing the contrast between the NM signal and background tissue [188]. They reported that not only were the SN and LC visible within the brainstem using a NM‐sensitive sequence, but that the signal in the said regions was significantly reduced in PD compared to healthy controls [188]. Furthermore, a postmortem analysis of PD and DLB formalin‐fixed midbrains found that NM‐MRI intensity in the SNc was highly correlated with the number of dopaminergic neurons present [189]. Thus, NM‐MRI is considered a promising proxy marker of dopaminergic neuron concentration.

In support of this, several subsequent studies have demonstrated that NM‐MRI has utility for characterising SNc degeneration in PD [190, 191], with a consensus that the NM‐MRI signal in the SNc is significantly reduced in PwP compared to healthy controls (see, for example, a systematic review of 12 studies, [40]). In fact, NM‐MRI may play a key role in improving diagnosis, with *de novo* PD patients showing reduced signal in the lateral, medial and central SNc compared to controls [192]. Of note, previous work investigating maximal SN length has reported significant differences between *de novo* PD and controls with a specificity and sensitivity of 70% and 83.3%, respectively [193]. This suggests that NM‐MRI can act as a marker of NM content within the SNc, even at early stages of PD. Furthermore, NM‐MRI can characterise progressive deterioration of the SNc. In line with this, longitudinal studies have reported that NM‐MRI is sensitive enough to capture progressive signal loss in the SNc over time, with this loss correlated with both disease duration and disease severity [194, 195].

**Figure 2:**
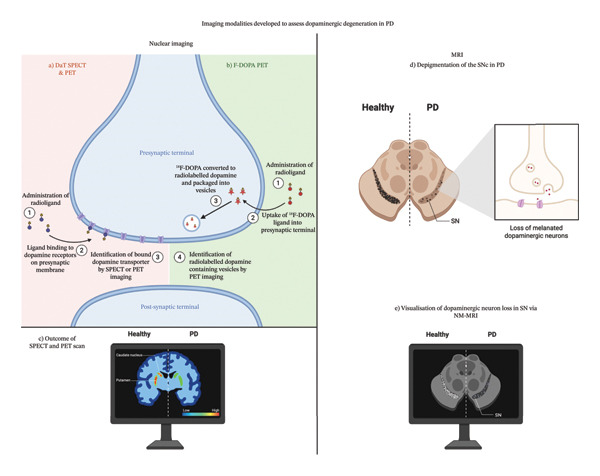
Several assessments to measure dopamine changes in vivo in PD have been developed, including nuclear imaging techniques such as (a) DaT SPECT or PET and (b) F‐DOPA PET, with significantly lower binding being present in several regions of interest in PD relative to healthy controls (c). Similarly, (d) loss of neuromelanin resulting in the depigmentation of the SN throughout PD can be visualised via optimised MRI sequences (NM‐MRI) (e).

One major limitation in early NM‐MRI studies, as well as conventional MRI sequences, is that SNc reductions in PD are comparable to those seen in other parkinsonian disorders, namely, PSP, MSA and corticobasal degeneration (CBD). This highlights challenges in providing differential diagnosis between disorders, similar to those seen for DaT SPECT and F‐DOPA PET. Nevertheless, advances in NM‐MRI acquisition in recent years may allow these limitations to be overcome. In line with this, a study comparing MSA (*n* = 30) and PD (*n* = 10) determined that, using simple visual MRI analysis, the decreased unilateral SN and LC NM‐MRI signal was suggestive of PD (sensitivity = 90%, specificity = 81% and accuracy = 83%) [196]. Likewise, more profound NM‐MRI signal loss in the SNc and locus coeruleus (LC) has demonstrated promise in distinguishing PD tremor from essential tremor [197, 198]. Increased sensitivity has also translated to the ability to better characterise differential outcomes and progression across subtypes of PD. In one such study assessing 54 PwP, while both TD and rigid‐dominant PD had significantly lower SNc signal compared to controls, those classified as the postural instability‐gait disturbance (PIGD) subtype showed significantly lower contrast‐to‐noise ratios in the lateral SNc compared to those classified as the TD subtype, suggesting a more advanced pathology or rapid rate of progression in the PIGD group [199]. Furthermore, the SNc signal was able to distinguish between motor subtypes with a sensitivity and specificity of 76.5% and 78.6%, respectively [199]. Similarly, in a study of 32 PwP exploring freezing of gait (FOG), while SNc deterioration was present in individuals with and without FOG, LC degeneration was more profound in individuals with FOG [200]. Overall, not only does NM‐MRI show utility in differentiating PD from other parkinsonian disorders, but it may also provide an opportunity for improved characterisation of more individualised outcomes, translating to improved tracking and management. A summary of assessments developed to assess dopaminergic loss in PD is presented in Figure 2.

## 7. The Role of Loss of Other Neurotransmitters in PD

While the dopamine loss has remained a core focus of PD research, given its central role in the emergence of motor symptoms, it is important to acknowledge that neurotransmission changes are far more widespread than dopamine alone. In fact, multiple neurotransmitter systems are impacted in PD, including, but not limited to, glutamate, serotonin, acetylcholine and norepinephrine [201]. Of particular importance is norepinephrine, given its implications in the development of several nonmotor impairments in PD, such as autonomic dysfunction and cognitive complaints, as well as the neuroprotective benefits it confers to the dopaminergic system, which are lost throughout PD [202].

In support of this, a critical structure affected by PD pathology early on in the disease course is the LC, the primary source of norepinephrine for the CNS [203]. In fact, the neuronal loss within the LC of PwP is even more profound than that seen in the SNc [204], with Lewy body pathology disrupting the LC noradrenergic system prior to the dopaminergic nigrostriatal system, resulting in the emergence of various nonmotor symptoms, such as cognitive impairment, depression and sleep disorders in PD [205]. Therefore, in vivo assessment of the LC and the noradrenergic system may prove critical in earlier diagnosis, as well as the improved characterisation of nonmotor outcomes in PD.

### 7.1. Tools to Measure Norepinephrine Alterations as a Biomarker in PD

#### 7.1.1. NM‐Sensitive T1 MRI

In addition to investigating alterations within the SNc, as discussed above, NM‐MRI has also been explored in the LC. In individuals with PD, significantly lower NM content has been found in this region compared to healthy controls [188, 192, 206]. Furthermore, the NM‐MRI signal in the LC demonstrates relationships with several nonmotor symptoms of PD, even early on in the disease course. For example, *de novo* PwP with depressive symptoms display a significantly lower signal in the left LC compared to both controls and PwP without depressive symptoms [192], suggesting that depression in PD may be related to dysfunction within these pathways. Likewise, a study exploring *de novo* PD with MCI (*n* = 23) and without (*n* = 48) reported that the PD‐MCI group demonstrated lower contrast‐to‐noise ratios in the LC, which were negatively associated with Trail Making B scores [207], suggesting that the LC is critical for the presentation of cognitive impairment, particularly executive dysfunction, in PD. Links have also been demonstrated between the LC and the presentation of prodromal symptoms. In support of this, a comparative clinical study of clinical PD with and without RBD, as well as healthy controls, reported decreased NM‐MRI intensity in the LC, as well as widespread reductions in the noradrenergic PET ligand uptake in PD with RBD compared to healthy controls and PD without RBD, which correlated with the amount of REM sleep [208]. Despite these promising findings, however, several limitations and gaps are present in the current literature. For instance, many studies assessing NM‐MRI to characterise degeneration of the SNc and LC are cross‐sectional in nature, making it difficult to determine how neurodegeneration in the said regions corresponds to changes in clinical presentation over time.

#### 7.1.2. Noradrenergic Transporter Ligand

Several PET ligands have been developed to evaluate the noradrenergic system, with the most prominent of these being [^11^C]MRB, which targets the norepinephrine transporter (NET) [209]. NET is a protein found primarily on the membranes of noradrenergic neurons and is essential for the reuptake of norepinephrine following neurotransmission [210]. Consequently, in disease states characterised by the loss of noradrenergic neurons, NET expression decreases. [^11^C]MRB was developed and optimised within primate models, with early studies demonstrating that the regional signal seen postinjection is consistent with the known distribution of NET in the brain [210]. Furthermore, in a study in baboons comparing [^11^C]MRB to other NET ligands, including [^18^F]fluororeboxetine and (*R*)‐[^11^C]nisoxetine, as well as the serotonin transporter tracers [^11^C]oxaprotiline and [^11^C]lortalamine, [^11^C]MRB demonstrated a superior signal‐to‐noise ratio and binding specificity [211]. Therefore, [^11^C]MRB represents a promising *in vivo* tool to evaluate the integrity of the noradrenergic system and may even allow for earlier diagnosis of PD, given that the degeneration of LC occurs earlier than that seen in the nigrostriatal dopamine pathways in PD [212].

Despite this, however, PD‐specific [^11^C]MRB studies are scarce and represent an important avenue for future research. One recent study aimed to reduce PET acquisition time for [^11^C]MRB in idiopathic PD patients, demonstrating that a 30‐min acquisition was achievable [213]. This reduced acquisition time would significantly improve [^11^C]MRB studies in PD cohorts, reducing the overall discomfort, particularly if participants are asked to be off medication during the scan time. Therefore, [^11^C]MRB could be a useful tool that should be further validated in PD studies to probe beyond the nigrostriatal system.

## 8. The Role of Neuroinflammation in PD

Neuroinflammation is the innate inflammatory response of the central nervous system (CNS). It is a multisystem reaction, involving both activation of resident glial cells, primarily microglia and astrocytes (see review, [214]), and the recruitment of peripheral immune cells, with the overarching objective of restoring brain tissue to homeostatic levels following insult [215]. While neuroinflammation is vital, sometimes it becomes maladaptive and persists chronically [215]. Sustained neuroinflammation is highly detrimental to the CNS, as activated glial cells produce free radicals and neurotoxic substances [215]. Such chronic elevations of neuroinflammation have been widely reported in PD, with concentrations of several proinflammatory cytokines shown to be significantly upregulated in the CSF [216] and blood [217] of individuals with PD compared to healthy controls. In fact, it is hypothesised that oxidative stress and neurotoxicity due to chronic inflammation act as key players in brainstem nuclei degeneration in PD [218]. Given this, methods of accurately assessing inflammation may provide a novel diagnostic tool to diagnose and track progression in PD. While many methods are used to measure neuroinflammation in experimental models, to date, the most effective way to measure central neuroinflammation in vivo is via targeted PET tracers.

### 8.1. Tools to Measure Central Neuroinflammation as a Biomarker in PD

#### 8.1.1. TSPO Ligands

PET can be used to measure neuroinflammation via 18 kDa translocator protein (TSPO) ligands [219]. TSPO is a mitochondrial protein located on outer mitochondrial membranes and is primarily found in activated microglia [219], along with reactive astrocytes [220]. Under normal conditions, TSPO is not highly present in the brain; however, during neuroinflammation, it is highly upregulated [219]. Several TSPO ligands have been developed to date, as summarised in Table 3.

**Table 3:** Radioligands that bind to TSPO receptor sites as a marker of microglial activation and neuroinflammation.

Generation	Radioligand	Reference
1^st^	[^11^C]PK11195	[221–223]

2^nd^	[^11^C]SSR180575	[224]
	[^11^C]‐DPA‐713	[225]
	[^11^C]DAA1106	[226]
	[^18^F]FM‐PBR28	[227]
	[^18^F]PBR06	[228]

3^rd^	[^18^F]GE‐180	[229]

[^11^C]PK11195 was first radiolabelled in 1984 and subsequently integrated into many clinical and animal studies as a TSPO ligand used to measure neuroinflammation in vivo [221]. Of particular note, clinical imaging studies have shown significantly increased mean binding of [^11^C]PK11195 in the pons, BG and frontal regions in PD patients, compared to healthy controls [222]. Furthermore, [^11^C]PK11195 has been shown to be able to successfully detect microglial activation in individuals after a single moderate/severe TBI at varying time points, ranging from 11 months to 17 years, highlighting its potential to detect chronic levels of neuroinflammation [223]. However, [^11^C]PK11195 displays a low signal‐to‐noise ratio on account of nonspecific binding, with one study in baboons determining that, without correction for peripheral clearance, only 50% of tracer uptake was specific [230]. This corresponded to substantial challenges with interpretability and confidence in research findings.

To address these limitations, extensive research allowed for the development of the second‐generation radioligands, with improved viability for measuring neuroinflammation. These second‐generation ligands have heightened binding affinity and specificity to TSPO, on account of their indole‐ring structure [219]. A study using an acute neuroinflammation rat model, induced via 0.5 μL of (*R*, *S*)‐α‐amino‐3‐hydroxy‐5‐methyl‐4‐isoxazolepropionic stereotaxically injected into the right striatum, showed increased [^11^C]SSR180575 binding in the ipsilateral striatum, and an improved image contrast, compared to [^11^C]PK11195 [224]. In a study using one such second‐generation ligand, [^18^F]PBR06, to assess neuroinflammation in early PD (average disease duration = 2.5 years), it was reported that the standard uptake value ratio (regional uptake normalised against global brain uptake) in the putamen was significantly increased in PD compared to healthy controls, particularly on the ipsilateral side of motor onset [228]. Furthermore, [^18^F]PBR06 uptake was positively associated with the extent of dopaminergic denervation within the brainstem, measured using ^18^F‐FP‐DTBZ PET [228].

While the second‐generation ligands show improvement in binding affinity and specificity compared to the first‐generation ligands, it must be noted that binding affinity is significantly lower in a subset of individuals, due to a single nucleotide polymorphism in the TSPO gene present in 30% of the Caucasian population, but less so in Native American, African, Han Chinese and Japanese populations, resulting in the production of A147t TSPO, which destabilises the protein and ligand‐binding pocket, corresponding to reduced binding affinity [231]. To resolve this difficulty, [^18^F]GE‐180, a third‐generation TSPO radioligand has recently been developed. In addition to showing improved binding affinity for A147t TSPO, ^18^[F]GE‐180 is valued for its higher binding potential overall, allowing for increased signal‐to‐noise ratios [232]. Excitingly, preliminary data suggest that this ligand may improve assessment of neuroinflammation compared to previous generations. In line with this, a focal cerebral ischaemia rat model found that, compared to [^11^C]PK11195, [^18^F]GE‐180 uptake was 24% higher in the lesion and 18% lower in the contralateral healthy tissue, translating to a 20% in the signal‐to‐noise ratio [229]. To the best of our knowledge, research evaluating [^18^F]GE‐180 in clinical PD is yet to be reported. Therefore, future research needs to investigate [^18^F]GE‐180 in PD, with clinical trials aiming to do so currently underway (UMIN‐CTR–UMIN000030084).

## 9. The Role of Iron Accumulation in PD

Ferroptosis is a form of cell death, distinct from other forms, such as apoptosis and necrosis, arising from iron‐dependent lipid peroxidation and resulting in oxidative damage and eventual cell death (see review, [233]). Ferroptosis has been identified as a potential major driver of neuronal loss in PD [234]. This is supported by the fact that substantial iron accumulation is reported in PD, with Sofic and colleagues reporting that, in postmortem analysis of eight PD brains, total iron and ferric contents were 176% and 225% higher, respectively, in the PD SNc compared to age‐matched healthy controls [235]. Excitingly, advancements in imaging techniques allow for in vivo quantification of the regional iron content, which may show utility as a biomarker of PD.

### 9.1. Tools to Measure Iron Accumulation as a Biomarker in PD

#### 9.1.1. SWI and Quantitative Susceptibility Mapping (QSM)

SWI is an MRI modality developed to enhance visibility of certain types of tissue or abnormalities within the brain. This results from SWI being sensitive to different magnetic properties of various tissues, which are influenced by factors such as calcium, blood products and, most importantly for PD, iron [236]. It is widely recognised that, in PD, the regional iron content is significantly higher within both the SNc [237, 238] and the LC [238]. In a study of untreated PwP, significantly higher R2∗ values (a metric used in MRI research to quantify transverse relaxation rates, with higher values corresponding to the increased iron content [239]) were present in the lateral SNc in PD compared to healthy controls, which significantly predicted lateralised motor scores, a promising marker of disease severity [240].

Of note, regional iron accumulation in PD shows a progressive pattern, depending on the stage of PD, with iron deposition in the SNc predominately occurring in early stages [241]. Thus, SWI could be a useful tool in assessing disease progression. Importantly, however, SWI is unable to provide quantitative measures of magnetic susceptibility. This limitation has recently been addressed by the development of QSM. QSM has shown promise in identifying areas of high iron accumulation [242, 243], such as the SNc, with one study in individuals with multiple sclerosis (MS) demonstrating that QSM yielded superior sensitivity compared to R2∗ mapping, obtained via conventional MRI techniques [244].

Several studies have now assessed QSM and reported distinguishable iron accumulation in key brain ROIs, namely, subcortical BG structures, in individuals with PD [241, 245–249]. Of note, this pattern of accumulation can differentiate between those with PD and healthy controls, with significantly higher iron accumulation detected by QSM in the SNc in PD [245–247]. Furthermore, QSM displayed higher discriminatory capacity (AUC = 0.77) compared to either MRI transverse relaxation rate R2 (AUC = 0.72) or R2∗ (AUC = 0.65) values [247], suggesting that QSM may be more suitable for use in the diagnosis/prognosis of PD, compared to conventional MRI techniques. In line with this, QSM was able to detect abnormal SNc iron accumulation even in early PD [248], a major limitation of conventional T2 MRI sequences [239]. Furthermore, while many studies utilise MRI to visualise degeneration of the SNc in its entirety, a key focus in PD neuroimaging research involves delineating the SNc into nigrosomes to better characterise degeneration (see review, [250]). Of particular interest is nigrosome‐1, located within the dorsal region of the SN [251]. As Damier and colleagues established, the dopaminergic neurons that comprise nigrosome‐1 are particularly vulnerable and are amongst the earliest to degenerate in PD [252]. Thus, the absence of nigrosome‐1 could be a potentially early marker of PD. To date, however, there is a lack of standard protocols to visualise this territory [249]. Despite this limitation, an optimisation study demonstrated bilateral reductions in QSM‐derived values in the nigrosome‐1 of atypical parkinsonism and PD when compared to healthy controls [249].

QSM may also have utility for predicting the presentation and progression of disease symptoms in PD. Regional iron accumulation within the SNc highly correlated to the Hoehn–Yarr stage and UPDRS‐measured motor outcomes, with late PD demonstrating increased QSM values within the SNr, red nucleus (RN) and the GP, particularly the GPi, suggesting that these structures become involved during advanced stages of the disease [241]. In fact, a longitudinal study assessing 59 PwP over a 3‐year period found that baseline susceptibility of the SN, BG, RN, insular cortex and dentate gyrus predicted greater motor severity at a 3‐year follow‐up [253]. Beyond motor symptoms, QSM also demonstrated promise in predicting cognitive decline in this cohort, with baseline susceptibility of the right temporal cortex, nucleus basalis of Meynert and putamen predicting poorer cognitive performance at follow‐up [253]. This is in line with the results of another study, in which individuals with PD exhibited higher iron accumulation in their thalamic and hippocampal regions compared to healthy controls, which correlated negatively with their scores on the Mini‐Mental State Examination [254]. Interestingly, this susceptibility was significantly higher in Lewy body dementia compared to PD and healthy controls [254], with different iron deposition patterns also reported between MSA and PD [255], suggesting that QSM‐derived metrics are capable of distinguishing PD from other parkinsonian disorders, even those involving degeneration of dopaminergic systems, a limitation of techniques such as DaT SPECT and F‐DOPA PET.

As highlighted by Du and colleagues, however, QSM is not without its own limitations, including not being able to isolate the source of iron driving signal within the scan, or to identify where iron is located on a cellular level (neurons and glial cells, primarily microglia) within ROIs [256]. Furthermore, there is lack of standardisation relating to image and magnetic susceptibility acquisition, such as variation in the reference regions considered for relative measurement of magnetic susceptibility, limiting comparisons across studies [257]. Therefore, future work should focus on assessing QSM and its correlation with histopathological features in PD, as well as harmonisation of imaging and analytic protocols.

## 10. Conclusion

While several strides have been made in advancing PD diagnosis and management in recent years, substantial gaps remain. This is, at least in part, due to heavy reliance on clinical symptom presentation, particularly of the cardinal motor features of the disease, for diagnosis. This may contribute to the high rates of misdiagnosis seen in PD, leading to subsequent mistreatment. This has led to a push to redefine PD based on its biological presentation [7, 258], aided in large part by recent advances in neuroimaging. While a field still in its infancy, PET ligands show great promise in advancing the integration of neuroimaging into clinical settings for PD diagnosis. However, in isolation, such ligands lack specificity for PD and, therefore, need to be used in conjunction with additional markers to provide more confidence in differential diagnosis. These challenges may be mitigated by the development of a PET ligand specific for measuring α‐syn pathology, currently a major area of need for the field. Encouragingly, one such ligand, ^18^F ACI‐12589, is currently in development, but is not yet validated for clinical use [94]. Additionally, further research, particularly longitudinal studies tracking changes in signalling profiles over time, is critically needed before the widespread use of these radioligands in PD diagnosis and prognosis can be recommended. Another significant limitation of PET imaging is its considerable cost and the fact that it is not widely available. Thus, less expensive and more readily available imaging techniques, such as MRI, may offer a better alternative.

In recent years, specialised structural MRI sequences, including NM‐MRI and QSM, have delivered promising results. Nevertheless, they still present shortcomings that limit their clinical utility if used in isolation. Currently, there is a lack of automatic methods to segment PD‐relevant ROIs, such as the SNc [259, 260]. Therefore, manual segmentation is still the primary method used to delineate ROIs, which gives rise to subjectivity and variability between studies. Additionally, analysing subcortical structures in the inferior parts of the brain with structural sequences poses issues, due to pulsatile artefacts that are present in these deeper brain structures given their proximity to major blood vessels [261].

In addition to neuroimaging, direct measurement of biomarkers within biologic fluids, such as CSF or blood, offers exciting potential for revolutionising the diagnosis/prognosis of PD. Unfortunately, the measurement of such biomarkers in PD has traditionally only been available through CSF collection via highly invasive lumbar puncture. This limits its clinical utility, particularly for measuring disease progression over time. Importantly, recent advancements, such as the α‐syn SAA and skin biopsies, offer a new way forward. Early data have supported the utility of SAA and α‐syn skin biopsies for distinguishing between PD and healthy controls [42, 44], with the SAA showing promise for identifying which individuals with prodromal symptom presentation are likely to go on to develop PD [43], paving the way for earlier diagnosis [258]. While promising, significant additional work is needed to validate these tools prior to adoption into routine clinical practice.

Taken together, given that PD is a multifaceted disorder, it is likely that no one assessment used in isolation will be sufficient for diagnosis, or for tracking disease progression over time. Rather, the development and validation of a multimodal panel of assessments, incorporating several of the key pathological markers known to be associated with the disease, may be more suitable, leading to improved diagnostic confidence and advances in the understanding of the contribution of neuropathological changes to disease progression and outcome in PD.

## Author Contributions

A.M.: conceptualisation and writing–original draft; L.C.: writing–review and editing; I.B.: writing–review and editing and supervision; M.J.: writing–review and editing and supervision; and L.C‐P.: conceptualisation, writing–review and editing, and supervision.

## Funding

A.M. and L.C. were supported by an Australian Government Research Training Program Scholarship provided by the Department of Education and Training, Canberra, Australia. This work was supported by grants to L.C‐P. and I.B. by the Medical Research Future Fund (Grant No. 2020/MRF1202188), James and Diana Ramsay Foundation and Neurosurgical Research Foundation.

Open access publishing facilitated by Adelaide University, as part of the Wiley‐Adelaide University agreement via the Council of Australasian University Librarians.

## Conflicts of Interest

The authors declare no conflicts of interest.

## Data Availability

No data were used for the research described in the article.
